# Cross-cultural adaptation of the Iranian Voice Quality of Life Profile into Brazilian Portuguese

**DOI:** 10.1590/2317-1782/20232023023en

**Published:** 2024-05-31

**Authors:** Joyra da Silva Carrer, Fabiana Zambon, Ali Dehqan, Vanessa Veis Ribeiro, Mara Behlau

**Affiliations:** 1 Centro de Estudos da Voz – CEV - São Paulo (SP), Brasil.; 2 Sindicato dos Professores de São Paulo – SINPRO-SP - São Paulo (SP), Brasil.; 3 Zahedan University of Medical Sciences - Zahedan, Iran.; 4 Universidade de Brasília – UnB - Brasília (DF), Brasil.; 5 Universidade Federal de São Paulo – UNIFESP - São Paulo (SP), Brasil.

**Keywords:** Voice, Dysphonia, Protocols, Translation, Speech Pathology, Self-assessment

## Abstract

**Purpose:**

To cross-culturally adapt the Voice Quality of Life Profile (IVQLP) into Brazilian Portuguese (BP).

**Methods:**

The cross-cultural adaptation process was performed in five stages: translation of the IVQLP into BP by three native BP experts fluent in American English; preparation of a consensus version; back-translation by a native American English expert fluent in BP; analysis by a committee of five experts and preparation of the final version of the instrument in BP, which was named IVQLP-Br; and pre-testing. The IVQLP-Br aims to assess the impacts of the voice more comprehensively, encompassing various areas of an individual’s life. It has 43 items and a five-level response key. For the pre-test, the alternative “not applicable” was added as a response option. Thirty-six adults with self-reported risk of dysphonia participated in the pre-test.

**Results:**

In the translation stage, ten items were modified, and during the back-translation, 15 items required adjustments. No questions required reformulation after the application of the IVQLP-Br in the target population, because the option “not applicable” appeared in 12 responses without statistical significance.

**Conclusion:**

The version of the IVQLP translated into BP, named the IVQLP-Br, exhibited cross-cultural equivalence and was administrable for a more detailed analysis of the impact of the voice in different domains of an individual’s life. After validation, the IVQLP-Br will be able to contribute both to clinical practice and to research with BP speakers.

## INTRODUCTION

Any difficulty or deviation in vocal emission that hinders the natural production of the voice can be called dysphonia^(1)^. The voice is multidimensional; therefore, dysphonia can impact various aspects of an individual's quality of life, including physical, functional, emotional, or cultural aspects. It can cause limitations in interpersonal communication, stress, and anxiety, as well as social isolation, depending on the degree of vocal and emotional impairment^(2)^.

Due to the importance of the vocal self-perception dimension in dysphonia cases, ASHA recommends five procedures for a multidimensional voice assessment, including the individual's self-evaluation regarding their voice problem^(3)^. Furthermore, both physicians and speech-language pathologists in vocal clinics suggest multidimensional assessment to provide a better diagnosis^(4)^. Data from vocal self-assessment cannot be obtained only through analyses performed by the clinician^(5)^; thus, the self-assessment dimension must be considered. Self-assessment involves individuals evaluating various aspects of a problem using instruments designed for self-perception analysis, where the items correspond to the specific objectives that are being evaluated and are commonly referred to as skills or constructs^(6)^.

The application of vocal self-assessment instruments is an important, quick, and easily applicable strategy to understand the impact of a potential vocal problem from the patient's perspective. The results can help estimate the patient's adherence to treatment^(7)^ and broaden their perception of their voice problem. These protocols also enable speech-language pathologists to gather a set of valid and reliable measures that can be used alongside other procedures comprising multidimensional voice assessment to observe the patient's progress.

Quality of life is a construct of significant relevance in the field of voice assessment, which can be quantified using self-assessment instruments. It is defined as the individual's perception of their life situation within the context of their culture and values, regarding their goals, expectations, standards, and concerns^(8)^. This definition establishes interconnections between the environment and various factors including physical, psychological, level of independence, social relationships, and personal beliefs^(9)^.

Due to the complexity and subjectivity of this concept, the vocal clinic needs instruments that investigate how different aspects of the clinical condition^(10)^ may impact the individual's quality of life. In Brazilian Portuguese, there is currently only one validated instrument to analyze the voice-related quality of life, the V-RQOL (Voice-Related Quality of Life)^(5)^.

A recent development in the field is the instrument known as the *Iranian Voice Quality Of Life Profile* (IVQLP)^(2,11)^. It originates from Iran and consists of 43 items divided into three domains: physical (items 1 to 5), which refers to the individual's difficulty in voice use; emotional (items 6 to 26), which refers to the emotional state resulting from voice use; and functional regarding work, daily communication, and social activities (items 27 to 43), items related to voice use in environments that require more effective communication. The IVQLP is a validated self-assessment protocol that is considered to be more comprehensive in assessing the impact of dysphonia on the patient's quality of life. This facilitates a nuanced understanding of how voice issues affect various aspects of life quality, enhancing both clinical management and scientific research.

The procedures of translation, adaptation, and validation of instruments have been significantly growing in the field of Speech-Language Pathology in Brazil. However, the process must be systemized and follow methodological guidelines. To perform validation, it is essential to go through the previously mentioned stages and to use international guidelines that ensure the effective acquisition of psychometric or clinical metric properties of the test^(12)^.

When it comes to applying an instrument in a new language and culture, the literature offers various recommendations. The international recommendations from the Scientific Advisory Committee (SAC) of Medical Outcome Trust^(13)^ are widely accepted in the voice field. The SAC recommends that cross-cultural adaptation follows stages including translation, synthesis, back-translation, expert committee review, and pre-testing.

Therefore, for the IVQLP to be used in Brazil, it is necessary to translate and cross-culturally adapt it to Brazilian Portuguese, following the SAC's recommendations. Following this stage, the validation process begins, which also relies on the SAC's attributes: conceptual and measurement model; reliability; validity; interpretability; respondent and administrative demand; alternative forms; and cultural and language adaptations^(6)^.

It is known that other instruments, such as the V-RQOL^(5)^ and the Vocal Handicap Index – VHI^(14)^, are widely accepted, validated, and used in many languages. However, these were pioneering protocols, developed earlier and proposed without the scientific rigor now used in modern instruments such as the IVQLP. Moreover, the adaptation process marks the initial phase of this instrument validation, offering speech-language pathologists a novel clinical option. This allows for an expanded analysis, delving into additional dimensions of voice-related impact on quality of life and providing further insights. Consequently, it is essential to undertake a cross-cultural adaptation of the IVQLP into Brazilian Portuguese, followed by its validation. This is crucial as it enables a more comprehensive analysis of the impact of voice across various domains of individuals' lives. This contributes significantly to both clinical practice and research efforts.

Thus, this study aimed to cross-culturally adapt the IVQLP instrument to Brazilian Portuguese.

## METHODS

This is a cross-sectional observational study. The present research was approved by the Research Ethics Committee of the Federal University of Sergipe / *Universidade Federal do Sergipe* (CAAE:47463021.9.0000.5546 from 01/10/2021). All participants signed the Informed Consent Form (ICF).

The procedures followed international recommendations from the SAC of Medical Outcome Trust^(13)^. The study steps included translation, synthesis, back-translation, expert committee review, and pre-testing.

### Translation

The IVQLP was translated into Brazilian Portuguese by three Brazilian speech-language pathologists, voice specialists, native speakers of Brazilian Portuguese, and fluent in American English. The translators were instructed to perform conceptual translation according to the cultural aspects of the target language.

### Synthesis

A synthesized version was created based on the translations, considering the consensus among the three versions. Any discrepancies were resolved by the authors.

### Back-translation

The synthesized version in Brazilian Portuguese was back-translated by a speech-language pathologist, voice specialist, native speaker of American English, and fluent in Brazilian Portuguese.

### Expert Committee Review

Five Brazilian speech-language pathologists, voice specialists, native speakers of Brazilian Portuguese, and fluent in American English, took part in the expert committee. The committee included professionals who did not participate in the previous stages.

The back-translated version was initially compared to the instrument's original English version to identify possible conceptual inconsistencies. The issues identified were discussed, and modifications were made as necessary, resulting in a final Brazilian Portuguese version.

### Pre-testing

The final version of the instrument in Brazilian Portuguese was administered to individuals representing the target population. For the pre-testing, individuals from the target population answered to the final translated and adapted version of the IVQLP in Brazilian Portuguese. This instrument has 43 items and a four-point Likert scale response key, ranging from “none, not a problem” (1) to “a lot” (4). For this pre-testing, the response key included the option “not applicable,” to identify items not understood or not suitable for the target population or the Brazilian culture.

Participants from the pre-testing group were recruited through dissemination on social media and via the institutional email, which contained an invitation to participate in the research along with the participation link. Data collection was conducted online using Google Forms; there was a total of 52 respondents.

For the sample selection, volunteers answered a screening questionnaire with direct questions related to the inclusion and exclusion criteria (Appendix A), such as the presence of vocal complaints and neurological or cognitive alterations. They also responded to the validated Brazilian Portuguese version of the Vocal Handicap Index (VHI-10)^(14)^. Participants who reported neurological or cognitive impairments hindering their comprehension of the research items were excluded. The final pre-testing sample included individuals aged from 18 to 60 years old, of Brazilian nationality, and at risk for dysphonia (VHI-10 with a score above the cutoff point of 7.5 points), totaling 36 participants.

The VHI-10 is a self-assessment protocol that evaluates the self-perceived impact of vocal alteration. It has 10 statements and a 5-level response key, ranging from “never” (0) to “always” (4). The instrument yields a single total score, calculated by the simple sum of responses to its items, ranging from 0 (no handicap) to 40 (maximum handicap). The instrument cutoff point is 7.5 points; participants scoring 8 or more points are considered at risk for dysphonia^(14)^.

The pre-testing data analysis was conducted using SPSS 25.0 software. A comparison was made between the proportion of non-applicable responses and the proportion of individuals who selected standard options in the instrument's response key (responses on the Likert scale from 1 to 4) using the Two-Proportion Z-test. A p-value of <0.05 was considered significant.

## RESULTS

The process of translation, synthesis, back-translation, and expert committee review are presented in Chart 1. Consensus was achieved on 33 items during the translation phase. The items that did not achieve consensus were: 3, 12, 17, 25, 29, 31, 36, 37, 38, and 43. These items were revised by the authors to ensure language clarity and ease the understanding of respondents.

**Chart 1:** Process of translation, synthesis, back-translation, and expert committee review of the Voice Quality of Life Profile (IVQLP)

**Item**	**English Version**	**T1**	**T2**	**T3**	**Synthesis**	**Back-translation**	**Expert Committee Review**	**SLP Committee: cultural and language adaptations**
		**English-Portuguese**	**English-Portuguese**	**English-Portuguese**				
**Title**	Iranian Voice-oriented Quality of Life Profile (IVQLP)	Perfil de Qualidade de vida relacionado à voz	Perfil de Qualidade de vida relacionado à voz	Perfil de Qualidade de vida relacionado à voz	Perfil de Qualidade de vida relacionado à voz	Voice Quality of life profile	Perfil de Qualidade de vida relacionado à voz	Perfil de Qualidade de vida relacionado à voz
**Instruction**	Please answer the following questions depending on the extent of the problem you face. Use the following scale	T1: Responda às seguintes perguntas de acordo com a extensão do problema que você enfrenta. Use a seguinte escala para avaliar a dimensão do problema:	T2: Por favor, responda as questões de acordo com o seu problema de voz. Use a escala a seguir para quantificar a extensão do seu problema:	T3: Responda às seguintes perguntas dependendo da magnitude do problema que você enfrenta. Use a seguinte escala para quantificar o tamanho do seu problema:	Responda às seguintes perguntas dependendo do tamanho do seu problema Use a seguinte escala:	Respond to the following questions according to the size of your problem using the following scale:	Responda às seguintes perguntas dependendo do tamanho do seu problema. Use a seguinte escala:	Responda às seguintes perguntas dependendo do tamanho do seu problema. Use a seguinte escala:
	for rating the amount of the problem:							
**Response Key**	1 = None, not a problem	T1: 1 = Não, não é um problema	T2: 1 = Nenhum, não é um problema	T3: 1 = Nenhum, não é um problema	1 = Nenhum, não é um problema	1 = It is not a problem	1 = Nenhum, não é um problema	1 = Nenhum, não é um problema
	2 = A small amount	2 =pequena dimensão	2 = em pequena quantidade	2=Pequeno	2 = é um problema pequeno	2= It is a mild problem	2 = é um problema pequeno	2 = é um problema pequeno
	3 = A moderate (medium) amount	3=modera-da (média) dimensão	3 = Em uma quantidade moderada (média)	3=Modera-do	3= é um problema moderado	3= It is a moderate problem	3= é um problema moderado	3= é um problema moderado
	4 = A lot	4 = grande dimensão	4 = Bastante	4 = Muito grande	4 = é um problema grande	4= It is a severe problem	4 = é um problema grande	4 = é um problema grande
							
**1**	I have trouble in speaking long by phone.	T1: Tenho problemas para falar ao telefone por um longo período	T2: Eu tenho problema para falar muito ao telefone	T3: Eu tenho problemas para falar muito tempo ao telefone	Eu tenho problema para falar muito ao telefone	I have trouble speaking on the phone for too long	Eu tenho problema para falar muito ao telefone.	Eu tenho problemas para falar muito ao telefone.

**2**	I have trouble in speaking loudly.	T1: Tenho problemas para falar alto	T2: Eu tenho problema para falar alto	T3: Eu tenho problemas para falar forte	Eu tenho problema para falar alto	I have trouble when speaking loudly	Eu tenho problema para falar alto.	Eu tenho problemas para falar alto.	

**3**	I have headache while speaking.	T1: Tenho dores de cabeça enquanto falo	T2: Eu tenho dor de cabeça enquanto falo	T3: Eu tenho dor de cabeça enquanto falo	Eu tenho dor de cabeça quando falo	I have headache when I speak	Eu tenho dor de cabeça quando falo.	Eu tenho dor de cabeça quando eu falo.	

**4**	I have trouble in keeping my voice in speaking.	T1: Tenho dificuldade em manter a voz enquanto falo	T2: Eu tenho problema para manter minha voz estável enquanto falo	T3: Eu tenho problemas para manter minha voz enquanto falo	Eu tenho problema para manter minha voz estável quando falo	I have trouble maintaining a stable voice when speaking	Eu tenho problema para manter minha voz estável quando falo.	Eu tenho problemas para manter a minha voz quando eu falo.	

**5**	My voice sometimes is good and sometimes goes wrong.	T1: Às vezes minha voz é boa e às vezes é ruim	T2: Minha voz às vezes é boa e às vezes sai ruim	T3: A minha voz às vezes é boa e às vezes é ruim	A minha voz às vezes é boa e às vezes é ruim	Sometimes my voice is good and sometimes its bad	A minha voz às vezes é boa e às vezes é ruim.	A minha voz às vezes está boa e às vezes ruim.	

**6**	I get nervous.	T1: Fico nervoso (a)	T2: Eu fico nervosa	T3: Eu fico nervoso	Eu fico nervoso (a)	I get nervous.	Eu fico nervoso (a).	Por causa da minha voz, eu fico nervoso (a).	

**7**	I feel to annoy others.	T1: Sinto que perturbo outras pessoas	T2: Eu sinto que incomodo as outras pessoas	T3: Eu sinto que irrito os outros	Eu sinto que incomodo os outros	I feel I annoy other people	Eu sinto que incomodo os outros	Por causa da minha voz, eu sinto que incomodo os outros.	

**8**	I have trouble in finding friends.	T1: Tenho dificuldade para fazer amigos	T2: Eu tenho dificuldade em encontrar amigos	T3: Eu tenho problemas em encontrar amigos	Eu tenho dificuldade para fazer amigos	I have trouble making friends	Eu tenho dificuldade para fazer amigos	Por causa da minha voz, eu tenho dificuldades para fazer amigos.	

**9**	I lost my self-confidence.	T1: Eu perdi minha autoconfiança	T2: Eu perdi a minha autoconfiança	T3: Eu perdi minha autoconfiança	Eu perdi minha autoconfiança	I lost my self-confidence	Eu perdi minha autoconfiança.	Por causa da minha voz, eu perdi a autoconfiança.	

**10**	I have trouble in self-expression.	T1: Tenho problema para me expressar	T2: Eu tenho problemas para me expressar	T3: Eu tenho problemas para me expressar	Eu tenho problemas para me expressar	I have trouble in expressing myself	Eu tenho problemas para me expressar.	Por causa da minha voz, eu tenho problemas para expressar quem eu sou.	

**11**	I have trouble in expressing my ideas.	T1: Tenho dificuldade para expressar minhas ideias	T2: Eu tenho problemas para expressar as minhas ideias	T3: Eu tenho problemas para expressar minhas ideias	Eu tenho problemas para expressar minhas ideias	I have trouble in expressing my ideas	Eu tenho problemas para expressar minhas ideias.	Por causa da minha voz, eu tenho problemas para expressar minhas ideias.	

**12**	I am embarrassed	T1: Fico constrangido (a)	T2: Eu estou envergonhado	T3: Eu estou envergonhado	Eu fico com vergonha	I get embarrassed	Eu fico com vergonha.	Por causa da minha voz, eu fico com vergonha.	

**13**	I lost my calmness.	T1: Perco minha calma	T2: Eu perdi a minha calma	T3: Eu perdi a minha calma	Eu perco minha calma	I lose my calmness	Eu perco minha calma.	Por causa da minha voz, eu perco a calma.	

**14**	I am ridiculed by others.	T1: Sou ridicularizado (a) por outras pessoas	T2: Eu sou ridicularizado pelos outros	T3: Eu sou ridicularizado pelos outros	Eu sou ridicularizado pelos outros	I am ridiculed by other people	Eu sou ridicularizado pelos outros.	Por causa da minha voz, eu sou ridicularizado (a).	

**15**	I feel rejection and loneliness.	T1: Sinto rejeição e solidão	T2: Sinto rejeição e solidão	T3: Eu me sinto rejeitado e sozinho	Eu me sinto rejeitado (a) e sozinho (a)	I feel rejected and lonely.	Eu me sinto rejeitado (a) e sozinho (a).	Por causa da minha voz, eu me sinto rejeitado (a) e sozinho (a).	

**16**	I feel frustration.	T1: Sinto frustração	T2: Sinto frustração	T3: Eu me sinto frustrado	Eu me sinto frustrado (a)	I feel frustrated	Eu me sinto frustrado (a)	Por causa da minha voz, eu me sinto frustrado (a).	

**17**	I feel reluctance in responding to others.	T1: Sinto relutância em responder aos outros	T2: Sinto relutância para responder aos outros	T3: Eu sinto relutância em responder os outros	Eu resisto para responder aos outros	I am unwilling to answer to others	Eu resisto para responder aos outros	Por causa da minha voz, eu evito responder aos outros.	

**18**	I feel anxious.	T1: Me sinto ansioso (a)	T2: Sinto ansiedade	T3: Eu me sinto ansioso	Eu me sinto ansioso (a)	I feel anxious	Eu me sinto ansioso (a)	Por causa da minha voz, eu me sinto ansioso (a).	

**19**	I have no hope for the future.	T1: Não tenho esperança no futuro	T2: Não tenho esperança no futuro	T3: Eu não tenho esperança no futuro	Eu não tenho esperança no futuro	I have no hope in the future	Eu não tenho esperança no futuro.	Por causa da minha voz, eu não tenho esperança no futuro.	

**20**	I freeze up.	T1: Eu congelo	T2: Eu me sinto congelado	T3: Eu congelo	Eu congelo quando falo	I freeze when I speak	Eu congelo quando falo.	Por causa da minha voz, eu congelo quando falo.	

**21**	I feel humiliation.	T1: Me sinto humilhado (a)	T2: Eu me sinto humilhado	T3: Eu me sinto humilhado	Eu me sinto humilhado (a)	I feel humiliated	Eu me sinto humilhado (a)	Por causa da minha voz, eu me sinto humilhado (a).	

**22**	I feel depression	T1: Me sinto deprimido	T2: Sinto depressão	T3: Eu me sinto depressivo	Eu me sinto deprimido (a)	I feel depressed	Eu me sinto deprimido (a)	Por causa da minha voz, eu me sinto deprimido (a).	

**23**	I am more aggressive	T1: Estou mais agressivo (a)	T2: Eu estou mais agressivo	T3: Eu estou mais agressivo	Eu estou mais agressivo (a)	I am more aggressive	Eu estou mais agressivo (a)	Por causa da minha voz, eu estou mais agressivo (a).	

**24**	I am more fragile.	T1: Estou mais frágil	T2: Eu estou mais frágil	T3: Eu estou mais frágil	Eu estou mais frágil	I am more fragile	Eu estou mais frágil.	Por causa da minha voz, eu estou mais frágil.	

**25**	I feel inefficient.	T1: Me sinto ineficiente	T2: Eu me sinto ineficiente	T3: Eu me sinto ineficiente	Eu me sinto incapaz	I feel incapable	Eu me sinto incapaz	Por causa da minha voz, eu me sinto incapaz.	

**26**	I feel my opinions are not taken seriously by others.	T1: Sinto que os outros não levam minha opinião a sério	T2: Sinto que as minhas opiniões não são levadas a sério pelos outros	T3: Eu sinto que minha opinião não é levada a sério pelos outros	Eu sinto que os outros não levam minha opinião a sério	I feel the others do not consider my opinion seriously	Eu sinto que os outros não levam minha opinião a sério	Por causa da minha voz, eu sinto que os outros não levam as minhas opiniões a sério.	

**27**	Because of my voice, job performance was affected.	T1: Por causa da minha voz, minha performance no trabalho foi afetada	T2: Por causa da minha voz, minha performance no trabalho foi afetada	T3: Por causa da minha voz, o meu desempenho no trabalho foi afetado	O meu desempenho no trabalho foi afetado por causa da minha voz	My performance in work was affected	O meu desempenho no trabalho foi afetado por causa da minha voz.	Por causa da minha voz, meu desempenho no trabalho foi prejudicado.	

**28**	Because of my voice, I lost my job.	T1: Por causa da minha voz, perdi meu emprego	T2: Por causa da minha voz, eu perdi meu emprego	T3: Por causa da minha voz, eu perdi meu trabalho	Eu perdi meu emprego por causa da minha voz	I lost my job	Eu perdi meu emprego por causa da minha voz.	Por causa da minha voz, eu perdi meu trabalho.	

**29**	Because of my voice, I am earning less and it resulted in economic problems.	T1: Por causa da minha voz, estou ganhando menos dinheiro e isso me trouxe problemas econômicos	T2: Por causa da minha voz, minha renda diminuiu e isso me trouxe problemas econômicos	T3: Por causa da minha voz, estou ganhando menos e isso resultou em um problema econômico	Eu estou ganhando menos por causa da minha voz	I am earning less	Estou tendo prejuízo financeiro por causa da minha voz.	Por causa da minha voz, eu estou ganhando menos e tenho problemas financeiros.	

**30**	My job promotion encountered with problem.	T1: Minha promoção de trabalho não aconteceu	T2: Tive problemas para ser promovido	T3: Eu tive problemas para ser promovido no emprego	Eu tive problemas para ser promovido no emprego	I had trouble being promoted in my job	Eu tive problemas para ser promovido no emprego.	Por causa da minha voz, eu tive problemas para ser promovido no meu trabalho.	

**31**	Because of my voice, I lost many vocational positions.	T1: Por causa da minha voz, eu perdi muitas posições de trabalho	T2: Por causa da minha voz, perdi muitas posições de trabalho	T3: Por causa da minha voz, eu perdi muitas posições vocacionais	Eu perdi muitas oportunidades no meu trabalho por causa da minha voz	I lost job opportunities	Eu perdi oportunidades no meu trabalho por causa da minha voz.	Por causa da minha voz, eu perdi oportunidades no meu trabalho.	

**32**	Because of my voice, I dropped to low levels of job.	T1: Por causa da minha voz, meu cargo foi rebaixado	T2: Por causa da minha voz, minha posição de trabalho foi rebaixada	T3: Por causa da minha voz, eu diminuí meu nível de trabalho	Eu fui rebaixado no meu trabalho por causa da minha voz	I got demoted at my job	Eu fui rebaixado no meu trabalho por causa da minha voz.	Por causa da minha voz, eu fui rebaixado (a) no meu trabalho.	

**33**	I have trouble in communicating	T1: Tenho problemas para me comunicar	T2: Eu tenho dificuldade para me comunicar	T3: Eu tenho problemas para me comunicar	Eu tenho problemas para me comunicar	I have trouble communicating	Eu tenho problemas para me comunicar.	Por causa da minha voz, eu tenho problemas em me comunicar.	

**34**	I avoid speaking as much as possible.	T1: Faço de tudo para não falar	T2: Eu evito falar o máximo que posso	T3: Eu evito o máximo possível falar	Eu evito falar o máximo possível	I avoid speaking as most as I can	Eu evito falar o máximo possível.	Por causa da minha voz, eu evito falar sempre que possível.	

**35**	Others are misunderstood.	T1: Outros são mal compreendidos	T2: As pessoas não me compreendem bem	T3: Outros são mal compreendidos	As pessoas não me compreendem bem	People do not understand me well	As pessoas não me compreendem bem.	Por causa da minha voz, eu sou mal interpretado (a) pelos outros.	

**36**	Understandability of my voice is reduced.	T1: A compreensão da minha voz está reduzida	T2: A inteligibilidade da minha voz está reduzida	T3: A inteligibilidade a minha fala é reduzida	As pessoas têm dificuldade para entender o que eu falo	People have trouble in understanding what I say	As pessoas têm dificuldade para entender o que eu falo.	Por causa da minha voz, os outros têm dificuldades em entender o que eu falo.	

**37**	I am indifferent to asking in various statuses.	T1: Não faço mais perguntas	T2: Estou indiferente para questionar em várias situações	T3: Eu estou indiferente para perguntas em vários status	Eu evito fazer perguntas em várias situações	I avoid making questions in many situations	Eu evito fazer perguntas em várias situações.	Por causa da minha voz, eu evito fazer perguntas em várias situações.	

**38**	People ask me to speak louder.	T1: As pessoas me pedem para falar mais alto	T2: As pessoas pedem para eu falar mais alto	T3: As pessoas me pedem para falar com inteligibilidade	As pessoas pedem para eu falar mais claro	People ask me to speak louder	As pessoas pedem para eu falar mais alto.	Por causa da minha voz, as pessoas pedem para eu falar mais alto.	

**39**	People request me to speak more intelligibly.	T1: As pessoas me pedem para falar melhor	T2: As pessoas pedem para eu falar mais claro	T3: As pessoas me pedem para falar com inteligibilidade	As pessoas pedem para eu falar mais claro	People ask me to speak clearer	As pessoas pedem para eu falar mais claro.	Por causa da minha voz, as pessoas pedem para eu falar mais claro.	

**40**	I have trouble communicating in noisy environments.	T1: Tenho dificuldade em me comunicar em locais barulhentos	T2: Eu tenho problemas para me comunicar em ambientes ruidosos	T3: Eu tenho dificuldades para me comunicar em locais barulhentos	Eu tenho dificuldade para falar em locais barulhentos	I have trouble when speaking in noisy environments	Eu tenho dificuldade para falar em locais barulhentos.	Por causa da minha voz, eu tenho dificuldades de falar em locais barulhentos.	

**41**	I have trouble in participation in social activities.	T1: Tenho problemas para participar de atividades sociais	T2: Eu tenho problemas para participar de atividades sociais	T3: Eu tenho problemas para participar de atividades sociais	Eu tenho problemas para participar de atividades sociais	I have problems participating in social activities	Eu tenho problemas para participar de atividades sociais.	Por causa da minha voz, eu tenho problemas para participar de atividades sociais.	

**42**	I avoid of speaking in public.	T1: Evito falar em público	T2: Eu evito falar em público	T3: Eu evito falar em público	Eu evito falar em público	I avoid speaking in public	Eu evito falar em público.	Por causa da minha voz, eu evito falar em público.	

**43**	My partnership in family decisions is decreased.	T1: Minha participação nas decisões familiares diminuiu	T2: A minha participação nas decisões da família diminuiu	T3: Minha participação nas decisões familiares diminuiu	Eu participo menos nas decisões da minha família	I participate less in my family’s decisions	Eu participo menos nas decisões da minha família.	Por causa da minha voz, eu participo menos das decisões familiares.	


Caption: T1 = English-Portuguese translator number 1; T2 = English-Portuguese translator number 2; T3 = English-Portuguese translator number 3; Synthesis = Brazilian-Portuguese version of translations from T1 + T2 + T3; SLP: Speech-language pathologist

Following back-translation, during the expert committee analysis, revisions were required in 15 items: 4, 5, 10, 13, 14, 17, 27, 28, 29, 30, 33, 34, 35, 38, and 43. The pronoun “eu” (“I” in English) was added to all statements to avoid doubt or interpretation errors. In the original instrument, “Because of my voice” was only included before items 6 and 33. To enhance clarity and prevent misinterpretation, the expert committee included “Por causa da minha voz” (In English “Because of my voice”) at the beginning of items 6 to 43.

Thirty-six adults at risk for vocal disorders participated in the pre-testing. The mean age was 39.39 ± 13.15 years, with 25 women and 11 men. Of these participants, 32 (88.89%) reported vocal complaints, while 4 (11.11%) did not.

During the pre-testing phase, the “not applicable” option was chosen for items 4, 5, 6, 7, 12, 13, 14, 18, 32, and 34. However, the proportion of “not applicable” responses was significantly lower compared to the options in the traditional response key for all items (Table 1).

**Table 1:** Pre-testing analysis for the target population

**Item**	**NA**		**Traditional Response Key**		**p-value**
	**n**	**%**	**n**	**%**	
Eu tenho problemas para falar muito ao telefone	0	0.00	36	100.00	1.000
Eu tenho problemas para falar alto	0	0.00	36	100.00	1.000
Eu tenho dor de cabeça quando eu falo	0	0.00	36	100.00	1.000
Eu tenho problemas para manter a minha voz quando eu falo	2	5.56	34	94.44	0.002
A minha voz às vezes está boa e às vezes ruim	0	0.00	36	100.00	1.000
Por causa da minha voz, eu fico nervoso (a)	1	2.78	35	97.22	<0.001
Por causa da minha voz, eu sinto que incomodo os outros	0	0.00	36	100.00	1.000
Por causa da minha voz, eu tenho dificuldades para fazer amigos	0	0.00	36	100.00	1.000
Por causa da minha voz, eu perdi a autoconfiança	0	0.00	36	100.00	1.000
Por causa da minha voz, eu tenho problemas para expressar quem eu sou	0	0.00	36	100.00	1.000
Por causa da minha voz, eu tenho problemas para expressar minhas ideias	0	0.00	36	10000	1.000
Por causa da minha voz, eu fico com vergonha	0	0.00	36	100.00	1.000
Por causa da minha voz, eu perco a calma	0	0.00	36	100.00	1.000
Por causa da minha voz, eu sou ridicularizado (a)	0	0.00	36	100.00	1.000
Por causa da minha voz, eu me sinto rejeitado (a) e sozinho (a)	0	0.00	36	100.00	1.000
Por causa da minha voz, eu me sinto frustrado (a)	0	0.00	36	100.00	1.000
Por causa da minha voz, eu evito responder aos outros	0	0.00	36	100.00	1.000
Por causa da minha voz, eu me sinto ansioso (a)	1	2.78	35	97.22	<0.001
Por causa da minha voz, eu não tenho esperança no futuro	0	0.00	36	100.00	1.000
Por causa da minha voz, eu congelo quando falo	0	0.00	36	100.00	1.000
Por causa da minha voz, eu me sinto humilhado (a)	0	0.00	36	100.00	1.000
Por causa da minha voz, eu me sinto deprimido (a)	0	0.00	36	100.00	1.000
Por causa da minha voz, eu estou mais agressivo (a)	0	0.00	36	100.00	1.000
Por causa da minha voz, eu estou mais frágil	0	0.00	36	100.00	1.000
Por causa da minha voz, eu me sinto incapaz	0	0.00	36	100.00	1.000
Por causa da minha voz, eu sinto que os outros não levam as minhas opiniões a sério	0	0.00	36	100.00	1.000
Por causa da minha voz, meu desempenho no trabalho foi prejudicado	0	0.00	36	100.00	1.000
Por causa da minha voz, eu perdi meu trabalho	0	0.00	36	100.00	1.000
Por causa da minha voz, eu estou ganhando menos e tenho problemas financeiros	0	0.00	36	100.00	1.000
Por causa da minha voz, eu tive problemas para ser promovido no meu trabalho	0	0.00	36	100.00	1.000
Por causa da minha voz, eu perdi oportunidades no meu trabalho	0	0.00	36	100.00	1.000
Por causa da minha voz, eu fui rebaixado (a) no meu trabalho	1	2.78	35	97.22	<0.001
Por causa da minha voz, eu tenho problemas em me comunicar	0	0.00	36	100.00	1.000
Por causa da minha voz, eu evito falar sempre que possível	1	2.78	35	97.22	<0.001
Por causa da minha voz, eu sou mal interpretado (a) pelos outros	0	0.00	36	100.00	1.000
Por causa da minha voz, os outros têm dificuldades em entender o que eu falo	0	0.00	36	100.00	1.000
Por causa da minha voz, eu evito fazer perguntas em várias situações	0	0.00	36	100.00	1.000
Por causa da minha voz, as pessoas pedem para eu falar mais alto	0	0.00	36	100.00	1.000
Por causa da minha voz, as pessoas pedem para eu falar mais claro	0	0.00	36	100.00	1.000
Por causa da minha voz, eu tenho dificuldades de falar em locais barulhentos	0	0.00	36	100.00	1.000
Por causa da minha voz, eu tenho problemas para participar de atividades sociais	0	0.00	36	100.00	1.000
Por causa da minha voz, eu evito falar em público	0	0.00	36	100.00	1.000
Por causa da minha voz, eu participo menos das decisões familiares	0	0.00	36	100.00	1.000

Two-Proportion Z-test

Caption: NA = not applicable

The final version of the IVQLP, translated and adapted for Brazilian Portuguese, was named IVQLP-Br (Annex A), consisting of 43 items and a four-level response key.

## DISCUSSION

Self-assessment protocols have been widely used in clinical practice in the healthcare field. They serve to understand the impact of a possible vocal problem from the patient's perspective and to help estimate their treatment adherence^(2,5,7,11,14-16)^.

The cross-cultural adaptation of self-assessment instruments aims to ensure their suitability for use in the target population. Through this process, it is possible to address sociocultural differences between cultures and languages, rather than simply translating the instrument. The instrument adaptation and subsequent validation have been extensively employed in the Speech-Language Pathology field to provide instruments with good psychometric properties in Brazilian Portuguese for use in vocal clinics^(15,16)^.

The IVQLP is originally an Iranian protocol that has already been used in English. This protocol is regarded as more comprehensive than previously validated instruments in Brazilian Portuguese, likely due to its deeper analysis of a wider range of domains related to voice quality of life. It has items from the physical, emotional, and functional domains; the functional domain is subdivided into three subscales: functional regarding work, daily communication, and social activities. The IVQLP delves into the relationship between voice and emotional well-being, as well as voice and work-related issues, as observed in question 25, which states “*por causa da minha voz, eu me sinto incapaz*” (in the English version “I feel inefficient”), or question 30, “*por causa da minha voz, eu tive problemas para ser promovido no meu trabalho*” (in the English version “My job promotion encountered with problem”); thus, the protocol seeks to recognize in-depth the impact of voice on different lives domains, contributing to a therapeutic plan that is tailored-made to the patient's needs. Moreover, it seems to be an interesting instrument to be used in research with Brazilian Portuguese speakers.

For the Brazilian Portuguese translation and cross-cultural adaptation, three speech-language pathologists specializing in voice and fluent English conducted the translations of the instrument. Subsequently, a synthesis version was developed, and the authors resolved any discrepancies. Following this, a back-translation was conducted, during which the expert committee adjusted 15 items and added the phrase “*Por causa da minha voz*” (In English, free translation, “Because of my voice”) before questions 6 to 43 to clarify statements and avoid doubts or misinterpretations.

In the pre-testing phase, the “not applicable” option was selected twelve times, with 2 occurrences each in items 4 and 6, and one occurrence each in items 5, 7, 12, 13, 14, 18, 32, and 34. However, these numbers did not show a significantly higher proportion compared to the options in the traditional response key for any of the instrument items; therefore, no questions needed modification. Overall, the target population understood the 43 items and found them applicable in Brazilian Portuguese.

Thus, it was possible to produce a cross-culturally adapted version for Brazilian Portuguese of the IVQLP^(10,11)^, named IVQLP-Br. We believe that this instrument can contribute to the vocal clinic, providing useful information for case management. Although this protocol is longer than other questionnaires available for vocal clinics, such as the VHI^(14)^ and V-RQOL^(5)^, it offers a more extensive scope compared to the V-RQOL, which stands alone in addressing the concept of quality of life up to now. However, the present study only focused on cross-cultural adaptation; hence, further research is needed to validate the instrument, demonstrating its psychometric properties and confirming that IVQLP-Br is valid, reliable, and has good diagnostic accuracy in Brazilian Portuguese.

## CONCLUSION

The Brazilian Portuguese translated version of the IVQLP referred to as IVQLP-Br, has shown cross-cultural equivalence. Once all validation steps are finalized, it will serve for a deeper examination of the voice impact across various aspects of individuals' lives. This will significantly contribute to both clinical practice and research involving Brazilian Portuguese speakers.

## Appendix A QUESTIONNAIRE

Sex: ( ) Male ( ) Female

Age:

Do you have vocal complaints: ( ) Yes ( ) No

Do you have neurological alterations: ( ) Yes ( ) No

Do you have cognitive alterations: ( ) Yes ( ) No

## Annex A. Perfil de qualidade de vida relacionado à voz (IVQLP-Br)

Responda às seguintes perguntas dependendo do tamanho do seu problema. Use a seguinte escala:

1 = nenhum, não é um problema

2 = é um problema pequeno

3 = é um problema moderado

4 = é um problema grande

**Table:**

1.	Eu tenho problemas para falar muito ao telefone	1	2	3	4
2.	Eu tenho problemas para falar alto	1	2	3	4
3.	Eu tenho dor de cabeça quando eu falo	1	2	3	4
4.	Eu tenho problemas para manter a minha voz quando eu falo	1	2	3	4
5.	A minha voz às vezes está boa e às vezes ruim	1	2	3	4
6.	Por causa da minha voz, eu fico nervoso (a)	1	2	3	4
7.	Por causa da minha voz, eu sinto que incomodo os outros	1	2	3	4
8.	Por causa da minha voz, eu tenho dificuldades para fazer amigos	1	2	3	4
9.	Por causa da minha voz, eu perdi a autoconfiança	1	2	3	4
10.	Por causa da minha voz, eu tenho problemas para expressar quem eu sou	1	2	3	4
11.	Por causa da minha voz, eu tenho problemas para expressar minhas ideias	1	2	3	4
12.	Por causa da minha voz, eu fico com vergonha	1	2	3	4
13.	Por causa da minha voz, eu perco a calma	1	2	3	4
14.	Por causa da minha voz, eu sou ridicularizado (a)	1	2	3	4
15.	Por causa da minha voz, eu me sinto rejeitado (a) e sozinho (a)	1	2	3	4
16.	Por causa da minha voz, eu me sinto frustrado (a)	1	2	3	4
17.	Por causa da minha voz, eu evito responder aos outros	1	2	3	4
18.	Por causa da minha voz, eu me sinto ansioso (a)	1	2	3	4
19.	Por causa da minha voz, eu não tenho esperança no futuro	1	2	3	4
20.	Por causa da minha voz, eu congelo quando falo	1	2	3	4
21.	Por causa da minha voz, eu me sinto humilhado (a)	1	2	3	4
22.	Por causa da minha voz, eu me sinto deprimido (a)	1	2	3	4
23.	Por causa da minha voz, eu estou mais agressivo (a)	1	2	3	4
24.	Por causa da minha voz, eu estou mais frágil	1	2	3	4
25.	Por causa da minha voz, eu me sinto incapaz	1	2	3	4
26.	Por causa da minha voz, eu sinto que os outros não levam as minhas opiniões a sério	1	2	3	4
27.	Por causa da minha voz, meu desempenho no trabalho foi prejudicado	1	2	3	4
28.	Por causa da minha voz, eu perdi meu trabalho	1	2	3	4
29.	Por causa da minha voz, eu estou ganhando menos e tenho problemas financeiros	1	2	3	4
30.	Por causa da minha voz, eu tive problemas para ser promovido no meu trabalho	1	2	3	4
31.	Por causa da minha voz, eu perdi oportunidades no meu trabalho	1	2	3	4
32.	Por causa da minha voz, eu fui rebaixado (a) no meu trabalho	1	2	3	4
33.	Por causa da minha voz, eu tenho problemas em me comunicar	1	2	3	4
34.	Por causa da minha voz, eu evito falar sempre que possível	1	2	3	4
35.	Por causa da minha voz, eu sou mal interpretado (a) pelos outros	1	2	3	4
36.	Por causa da minha voz, os outros têm dificuldades em entender o que eu falo	1	2	3	4
37.	Por causa da minha voz, eu evito fazer perguntas em várias situações	1	2	3	4
38.	Por causa da minha voz, as pessoas pedem para eu falar mais alto	1	2	3	4
39.	Por causa da minha voz, as pessoas pedem para eu falar mais claro	1	2	3	4
40.	Por causa da minha voz, eu tenho dificuldades de falar em locais barulhentos	1	2	3	4
41.	Por causa da minha voz, eu tenho problemas para participar de atividades sociais	1	2	3	4
42.	Por causa da minha voz, eu evito falar em público	1	2	3	4
43.	Por causa da minha voz, eu participo menos das decisões familiares	1	2	3	4
