# Supplementary material for: Cross-cultural adaptation of the Iranian Voice Quality of Life Profile into Brazilian Portuguese
Source: Codas. 2024 May 31;36(3):e20230023. doi: 10.1590/2317-1782/20232023023en (PMC466997; doi:10.1590/2317-1782/20232023023en)
Supplement: Translation

# Translation: Adaptação transcultural para o Português Brasileiro do Perfil de Qualidade de Vida relacionado a Voz

**DOI:** 10.1590/2317-1782/20232023023pt

**Authors:** Joyra da Silva Carrer, Fabiana Zambon, Ali Dehqan, Vanessa Veis Ribeiro, Mara Behlau

**Affiliations:** 1 Centro de Estudos da Voz – CEV - São Paulo (SP), Brasil.; 2 Sindicato dos Professores de São Paulo – SINPRO-SP - São Paulo (SP), Brasil.; 3 Zahedan University of Medical Sciences - Zahedan, Iran.; 4 Universidade de Brasília – UnB - Brasília (DF), Brasil.; 5 Universidade Federal de São Paulo – UNIFESP - São Paulo (SP), Brasil.

**Keywords:** Voz, Disfonia, Protocolos, Tradução, Fonoaudiologia, Autoavaliação

## Abstract

**Objetivo:**

Traduzir e adaptar de forma transcultural o Iranian Voice Quality of Life Profile (IVQLP) para o português brasileiro (PB).

**Método:**

O processo de adaptação transcultural foi realizado por meio de cinco etapas: tradução do IVQLP para o PB por três especialistas nativos do PB e fluentes no inglês americano; elaboração de uma versão de consenso; retrotradução por um especialista nativo do inglês americano e fluente no PB; análise por um comitê de cinco especialistas e elaboração da versão final do instrumento em PB, denominado IVQLP-Br; e pré-teste. O IVQLP-Br tem o objetivo de avaliar os impactos da voz de uma forma mais abrangente, englobando vários domínios da vida dos indivíduos, apresenta 43 itens e uma chave de respostas de cinco pontos. Para o pré-teste foi acrescida como opção para o respondente a alternativa “não aplicável”. Participaram do pré-teste 36 indivíduos adultos com risco autorrelatado para disfonia.

**Resultados:**

Na etapa de tradução 10 itens foram modificados e na retrotradução, 15 itens necessitaram de ajustes. Nenhum item precisou ser reformulado após a aplicação na população-alvo, pois a opção “não aplicável” apareceu em doze respostas, porém, sem significância estatística.

**Conclusão:**

Conclui-se que a versão traduzida do IVQLP para o PB, denominado IVQLP-Br, apresentou equivalência transcultural e pode ser utilizada para uma análise mais detalhada do impacto da voz nos diferentes domínios da vida dos indivíduos. Após a validação, o IVQLP-Br poderá contribuir tanto para a prática clínica, quanto para pesquisas com falantes do PB.

## INTRODUÇÃO

Qualquer dificuldade ou desvio da emissão vocal que impeça a produção natural da voz pode ser denominado de disfonia^(1)^. A voz é multidimensional, e por isso, a disfonia pode impactar diversos aspectos da qualidade de vida dos indivíduos, sejam eles físicos, funcionais, emocionais ou culturais. Ela pode gerar limitações na comunicação interpessoal, estresse e ansiedade, além de isolamento social, dependendo do grau de comprometimento vocal e emocional^(2)^.

Devido a importância da dimensão de autopercepção vocal no quadro de disfonia, a ASHA recomenda cinco procedimentos de avaliação multidimensional da voz, fazendo parte deles a autoavaliação do indivíduo a respeito do seu problema de voz^(3)^. Além disso, tanto médicos como fonoaudiólogos na clínica vocal sugerem avaliação multidimensional para se compor um melhor diagnóstico do paciente^(4)^. Os dados da autoavaliação vocal não podem ser obtidos por meio de análises realizadas pelo clínico^(5)^ e, portanto, essa dimensão de avaliação não pode ser desprezada. A autoavaliação de diversos aspectos de um problema é procedimento realizado pelo próprio sujeito por meio de instrumentos de análise da autopercepção, com itens relacionados ao objetivo que se quer avaliar, denominados de habilidade ou construto^(6)^.

A aplicação de instrumentos de autoavaliação vocal é uma estratégia importante, rápida e de fácil aplicação para compreender o impacto de um possível problema vocal na perspectiva do paciente. O resultado pode ajudar a estimar como será a adesão do paciente ao tratamento^(7)^, além de ampliar a percepção do mesmo em relação ao seu problema de voz. Esses protocolos também permitem que os fonoaudiólogos coletem um conjunto de medidas válidas e confiáveis ​​que podem ser utilizadas em conjunto com os demais procedimentos que compõem a avaliação multidimensional da voz para observação da evolução do paciente.

Um dos construtos de grande relevância na área de voz que pode ser mensurado por meio de instrumentos de autoavaliação é a qualidade de vida. Ela é definida como a percepção do indivíduo de sua condição na vida, no contexto da cultura e sistema de valores nos quais ele vive e em relação aos seus objetivos, expectativas, padrões e preocupações^(8)^. Essa definição inter-relaciona o meio ambiente com aspectos físicos, psicológicos, nível de independência, relações sociais e crenças pessoais^(9)^.

Devido à complexidade e subjetividade desse conceito, faz-se necessário que na clínica vocal haja instrumentos que investiguem como diferentes aspectos do quadro clínico^(10)^ impactam na qualidade de vida do sujeito. Em português brasileiro há apenas um instrumento para análise da qualidade de vida relacionada a voz validado atualmente, o QVV – Qualidade de Vida em Voz^(5)^.

Recentemente surgiu um instrumento denominado *Iranian Voice Quality Of Life Profile* (IVQLP)^(2,11)^. Ele é de origem iraniana, composto por 43 itens, que se dividem nos fatores: físico (itens 1 a 5), que se refere a dificuldade ou não do indivíduo em utilizar a voz; emocional (itens 6 a 26), que diz respeito ao estado emocional decorrente ao uso da voz; e, funcional em relação ao trabalho, comunicação diária e atividades sociais (itens 27 a 43), itens relacionados ao uso da voz em ambientes que requerem uma comunicação mais efetiva. Trata-se de um protocolo de autoavaliação, validado, e que é considerado mais aprofundado na mensuração do impacto da disfonia na qualidade de vida do paciente, o que possibilita, a partir dos resultados, compreender com mais detalhes o impacto do problema de voz nos diferentes domínios que compõem a qualidade de vida, podendo contribuir na conduta clínica e em pesquisas científicas.

Os procedimentos de tradução, adaptação e validação de instrumentos vem crescendo de forma significativa na área da Fonoaudiologia no Brasil, porém ainda é necessário sistematizar o percurso metodológico. Para a realização da validação, é imprescindível passar pelas etapas citadas, bem como utilizar diretrizes internacionais que garantam a efetiva obtenção das propriedades psicométricas ou clinimétricas do teste^(12)^.

Para a aplicação de um instrumento em um novo idioma e cultura há diferentes recomendações na literatura.

As recomendações internacionais do Scientific Advisory Committee (SAC) of Medical Outcome Trust^(13)^ são as mais aceitas na área de Voz. O SAC recomenda que a adaptação transcultural seja composta pelas etapas de tradução, síntese, retrotradução, análise por um comitê de especialistas e pré-teste.

Dessa forma, para que seja possível utilizar o IVQLP no Brasil, faz-se necessário realizar sua tradução e adaptação transcultural para o português brasileiro, de acordo com as recomendações do SAC.

Após essa etapa de adaptação, inicia-se o processo de validação que também tem como base as etapas do SAC, sendo elas: modelo conceitual e de medida; confiabilidade; validade; sensibilidade; interpretabilidade; demanda de administração e resposta; modos alternativos de aplicação e adaptação linguística e cultural^(6)^.

Sabemos que outros instrumentos, como o QVV^(5)^ e o Índice de Desvantagem Vocal - IDV^(14)^ são amplamente aceitos, validados e utilizados em diversas línguas. Contudo, foram protocolos pioneiros, mais antigos e propostos sem o rigor científico atualmente presente nos instrumentos mais modernos, como o IVQLP. Além disso, a adaptação será o primeiro passo para que seja iniciado o processo de validação do instrumento, para que uma nova possibilidade de escolha clínica será dada ao fonoaudiólogo, que poderá contar com mais dimensões de análise com esse novo instrumento, explorando outros aspectos de influência de um problema de voz na qualidade de vida. Por essas razões, entende-se que o IVQLP mereça adaptação transcultural ao português brasileiro e posterior validação, por analisar de uma forma mais detalhada o impacto da voz nos diferentes domínios da vida dos indivíduos, além de contribuir tanto para a prática clínica, quanto para pesquisas.

Dessa forma, o objetivo deste estudo foi adaptar transculturalmente o instrumento IVQLP para português brasileiro.

## MÉTODO

Trata-se de um estudo observacional transversal. A presente pesquisa foi aprovada pelo Comitê de Ética em Pesquisa da Universidade Federal do Sergipe (CAAE:47463021.9.0000.5546 de 01/10/2021). Todos os participantes assinaram o Termo de Consentimento Livre e Esclarecido (TCLE).

Os procedimentos realizados seguiram as recomendações internacionais do *SACC of Medical Outcome Trust* ^(13)^. As etapas foram: tradução, síntese, retrotradução, análise por um comitê especialista e pré-teste.

### Tradução

O IVQLP foi traduzido para o português brasileiro por três fonoaudiólogos brasileiros, especialistas em voz, nativos do português brasileiro e falantes do inglês americano. Os tradutores foram orientados a realizarem a tradução conceitual de acordo com os aspectos culturais do idioma alvo.

### Síntese

Foi elaborada uma versão de síntese entre as traduções. Para isso considerou-se a concordância entre as três traduções. As discordâncias foram resolvidas pelos autores.

### Retrotradução

A versão síntese em PB foi retrotraduzida por um fonoaudiólogo, especialista em voz, nativo do inglês americano e fluente no português brasileiro.

### Análise do comitê de especialistas

Participaram do comitê de especialistas cinco fonoaudiólogos brasileiros, especialistas em voz, nativos do português brasileiro e falantes do inglês americano. O comitê foi composto por fonoaudiólogos especialistas em voz que não haviam participado das etapas anteriores.

A versão retrotraduzida foi inicialmente comparada à versão original em inglês do instrumento, a fim de verificar possíveis incoerências conceituais. Os problemas encontrados foram discutidos e, quando necessário, modificações foram realizadas, chegando-se a uma versão final em português brasileiro.

### Pré-teste

A versão final do instrumento em português brasileiro foi aplicada em indivíduos da população-alvo. Para o pré-teste, os indivíduos da população-alvo responderam a versão final traduzida e adaptada para o PB do IVQLP. Trata-se de um instrumento com 43 itens e uma chave de resposta em escala Likert de quatro pontos, que vai de não é um problema (1) a é um grande problema (4). Para o pré-teste foi acrescentada a chave da resposta a opção “não aplicável”, para identificação de itens não compreendidos ou não apropriados para a população-alvo ou para a cultura brasileira.

O recrutamento dos participantes para o pré-teste foi realizado por meio da divulgação em redes sociais e pelo e-mail institucional de um convite para participar da pesquisa que continha o link de participação. A coleta de dados foi realizada no formato *on-line* pelo *Google Forms*, onde houve 52 respondentes.

Para a seleção da amostra os voluntários responderam a um questionário amostral com perguntas diretas relacionadas aos critérios de inclusão e exclusão (Apêndice A) como a presença de queixa vocal, alterações neurológicas ou cognitivas, além de responderem também a versão validada em PB do Índice de Desvantagem Vocal (IDV-10)^(14)^. Os participantes que relataram nesse questionário alterações neurológicas ou cognitivas que não permitia compreender os itens do instrumento da pesquisa foram excluídos. Foram incluídos nessa etapa os participantes com idade entre 18 e 60 anos, nacionalidade brasileira, na faixa de risco para disfonia (IDV-10 com escore acima do ponto de corte de 7,5 pontos), totalizando 36 participantes.

O IDV-10 é um protocolo de autoavaliação que avalia a autopercepção do impacto de alteração vocal, composto por 10 itens e uma chave de resposta de 5 níveis, entre nunca (0) e sempre (4). O instrumento produz um escore total único, calculado por somatório simples das respostas de seus itens, podendo variar de 0 (nenhuma desvantagem) e 40 (desvantagem máxima). O instrumento possui um ponto de corte de 7,5 pontos, sendo que os participantes que têm escores de 8 ou mais pontos são considerados com risco para disfonia^(14)^.

A análise de dados do pré-teste foi realizada com o software SPSS 25.0. Foi realizada a comparação da proporção de respostas não-aplicáveis e da proporção de indivíduos que marcaram opções habituais da chave de resposta do instrumento (respostas na escala de likert de 1 a 4) com o Teste de Igualdade de Duas Proporções. O valor de p<0,05 foi considerado significante.

## RESULTADOS

O processo de tradução, síntese, retrotradução e análise do comitê de especialistas encontra-se no Quadro 1. Na síntese da tradução houve consenso em 33 itens. Os itens que não apresentaram consenso foram: 3, 12, 17, 25, 29, 31, 36, 37, 38 e 43. Esses itens foram ajustados pelos autores com o objetivo de se conseguir uma linguagem mais fácil e clara para os respondentes.

**Quadro 1:** Processo de tradução, síntese, retrotradução e análise do comitê de especialistas Voice Quality of Life Profile (IVQLP)

Item	Versão original em inglês	T1	T2	T3	Síntese	Retrotradução	Comitê de especialistas	Comitê de fonoaudiólogos: equivalência cultural e linguística
		Inglês-português	Inglês-português	Inglês-português				
Título	Iranian Voice-oriented Quality of Life Profile (IVQLP)	Perfil de Qualidade de vida relacionado à voz	Perfil de Qualidade de vida relacionado à voz	Perfil de Qualidade de vida relacionado à voz	Perfil de Qualidade de vida relacionado à voz	Voice Quality of life profile	Perfil de Qualidade de vida relacionado à voz	Perfil de Qualidade de vida relacionado à voz
Instrução	Please answer the following questions depending on the extent of the problem you face. Use the following scale	T1: Responda às seguintes perguntas de acordo com a extensão do problema que você enfrenta. Use a seguinte escala para avaliar a dimensão do problema:	T2: Por favor, responda as questões de acordo com o seu problema de voz. Use a escala a seguir para quantificar a extensão do seu problema:	T3: Responda às seguintes perguntas dependendo da magnitude do problema que você enfrenta. Use a seguinte escala para quantificar o tamanho do seu problema:	Responda às seguintes perguntas dependendo do tamanho do seu problema Use a seguinte escala:	Respond to the following questions according to the size of your problem using the following scale:	Responda às seguintes perguntas dependendo do tamanho do seu problema. Use a seguinte escala:	Responda às seguintes perguntas dependendo do tamanho do seu problema. Use a seguinte escala:
	for rating the amount of the problem:							
Chave de respostas	1 = None, not a problem	T1: 1 = Não, não é um problema	T2: 1 = Nenhum, não é um problema	T3: 1 = Nenhum, não é um problema	1 = Nenhum, não é um problema	1 = It is not a problem	1 = Nenhum, não é um problema	1 = Nenhum, não é um problema
	2 = A small amount	2 =pequena dimensão	2 = em pequena quantidade	2=Pequeno	2 = é um problema pequeno	2= It is a mild problem	2 = é um problema pequeno	2 = é um problema pequeno
	3 = A moderate (medium) amount	3=modera-da (média) dimensão	3 = Em uma quantidade moderada (média)	3=Moderado	3= é um problema moderado	3= It is a moderate problem	3= é um problema moderado	3= é um problema moderado
	4 = A lot	4 = grande dimensão	4 = Bastante	4 = Muito grande	4 = é um problema grande	4= It is a severe problem	4 = é um problema grande	4 = é um problema grande
							
1	I have trouble in speaking long by phone.	T1: Tenho problemas para falar ao telefone por um longo período	T2: Eu tenho problema para falar muito ao telefone	T3: Eu tenho problemas para falar muito tempo ao telefone	Eu tenho problema para falar muito ao telefone	I have trouble speaking on the phone for too long	Eu tenho problema para falar muito ao telefone.	Eu tenho problemas para falar muito ao telefone.

2	I have trouble in speaking loudly.	T1: Tenho problemas para falar alto	T2: Eu tenho problema para falar alto	T3: Eu tenho problemas para falar forte	Eu tenho problema para falar alto	I have trouble when speaking loudly	Eu tenho problema para falar alto.	Eu tenho problemas para falar alto.	

3	I have headache while speaking.	T1: Tenho dores de cabeça enquanto falo	T2: Eu tenho dor de cabeça enquanto falo	T3: Eu tenho dor de cabeça enquanto falo	Eu tenho dor de cabeça quando falo	I have headache when I speak	Eu tenho dor de cabeça quando falo.	Eu tenho dor de cabeça quando eu falo.	

4	I have trouble in keeping my voice in speaking.	T1: Tenho dificuldade em manter a voz enquanto falo	T2: Eu tenho problema para manter minha voz estável enquanto falo	T3: Eu tenho problemas para manter minha voz enquanto falo	Eu tenho problema para manter minha voz estável quando falo	I have trouble maintaining a stable voice when speaking	Eu tenho problema para manter minha voz estável quando falo.	Eu tenho problemas para manter a minha voz quando eu falo.	

5	My voice sometimes is good and sometimes goes wrong.	T1: Às vezes minha voz é boa e às vezes é ruim	T2: Minha voz às vezes é boa e às vezes sai ruim	T3: A minha voz às vezes é boa e às vezes é ruim	A minha voz às vezes é boa e às vezes é ruim	Sometimes my voice is good and sometimes its bad	A minha voz às vezes é boa e às vezes é ruim.	A minha voz às vezes está boa e às vezes ruim.	

6	I get nervous.	T1: Fico nervoso (a)	T2: Eu fico nervoso	T3: Eu fico nervoso	Eu fico nervoso (a)	I get nervous.	Eu fico nervoso (a).	Por causa da minha voz, eu fico nervoso (a).	

7	I feel to annoy others.	T1: Sinto que perturbo outras pessoas	T2: Eu sinto que incomodo as outras pessoas	T3: Eu sinto que irrito os outros	Eu sinto que incomodo os outros	I feel I annoy other people	Eu sinto que incomodo os outros	Por causa da minha voz, eu sinto que incomodo os outros.	

8	I have trouble in finding friends.	T1: Tenho dificuldade para fazer amigos	T2: Eu tenho dificuldade em encontrar amigos	T3: Eu tenho problemas em encontrar amigos	Eu tenho dificuldade para fazer amigos	I have trouble making friends	Eu tenho dificuldade para fazer amigos	Por causa da minha voz, eu tenho dificuldades para fazer amigos.	

9	I lost my self-confidence.	T1: Eu perdi minha autoconfiança	T2: Eu perdi a minha autoconfiança	T3: Eu perdi minha autoconfiança	Eu perdi minha autoconfiança	I lost my self-confidence	Eu perdi minha autoconfiança.	Por causa da minha voz, eu perdi a autoconfiança.	

10	I have trouble in self-expression.	T1: Tenho problema para me expressar	T2: Eu tenho problemas para me expressar	T3: Eu tenho problemas para me expressar	Eu tenho problemas para me expressar	I have trouble in expressing myself	Eu tenho problemas para me expressar.	Por causa da minha voz, eu tenho problemas para expressar quem eu sou.	

11	I have trouble in expressing my ideas.	T1: Tenho dificuldade para expressar minhas ideias	T2: Eu tenho problemas para expressar as minhas ideias	T3: Eu tenho problemas para expressar minhas ideias	Eu tenho problemas para expressar minhas ideias	I have trouble in expressing my ideas	Eu tenho problemas para expressar minhas ideias.	Por causa da minha voz, eu tenho problemas para expressar minhas ideias.	

12	I am embarrassed	T1: Fico constrangido (a)	T2: Eu estou envergonhado	T3: Eu estou envergonhado	Eu fico com vergonha	I get embarrassed	Eu fico com vergonha.	Por causa da minha voz, eu fico com vergonha.	

13	I lost my calmness.	T1: Perco minha calma	T2: Eu perdi a minha calma	T3: Eu perdi a minha calma	Eu perco minha calma	I lose my calmness	Eu perco minha calma.	Por causa da minha voz, eu perco a calma.	

14	I am ridiculed by others.	T1: Sou ridicularizado (a) por outras pessoas	T2: Eu sou ridicularizado pelos outros	T3: Eu sou ridicularizado pelos outros	Eu sou ridicularizado pelos outros	I am ridiculed by other people	Eu sou ridicularizado pelos outros.	Por causa da minha voz, eu sou ridicularizado (a).	

15	I feel rejection and loneliness.	T1: Sinto rejeição e solidão	T2: Sinto rejeição e solidão	T3: Eu me sinto rejeitado e sozinho	Eu me sinto rejeitado (a) e sozinho (a)	I feel rejected and lonely.	Eu me sinto rejeitado (a) e sozinho (a).	Por causa da minha voz, eu me sinto rejeitado (a) e sozinho (a).	

16	I feel frustration.	T1: Sinto frustração	T2: Sinto frustração	T3: Eu me sinto frustrado	Eu me sinto frustrado (a)	I feel frustrated	Eu me sinto frustrado (a)	Por causa da minha voz, eu me sinto frustrado (a).	

17	I feel reluctance in responding to others.	T1: Sinto relutância em responder aos outros	T2: Sinto relutância para responder aos outros	T3: Eu sinto relutância em responder os outros	Eu resisto para responder aos outros	I am unwilling to answer to others	Eu resisto para responder aos outros	Por causa da minha voz, eu evito responder aos outros.	

18	I feel anxious.	T1: Me sinto ansioso (a)	T2: Sinto ansiedade	T3: Eu me sinto ansioso	Eu me sinto ansioso (a)	I feel anxious	Eu me sinto ansioso (a)	Por causa da minha voz, eu me sinto ansioso (a).	

19	I have no hope for the future.	T1: Não tenho esperança no futuro	T2: Não tenho esperança no futuro	T3: Eu não tenho esperança no futuro	Eu não tenho esperança no futuro	I have no hope in the future	Eu não tenho esperança no futuro.	Por causa da minha voz, eu não tenho esperança no futuro.	

20	I freeze up.	T1: Eu congelo	T2: Eu me sinto congelado	T3: Eu congelo	Eu congelo quando falo	I freeze when I speak	Eu congelo quando falo.	Por causa da minha voz, eu congelo quando falo.	

21	I feel humiliation.	T1: Me sinto humilhado (a)	T2: Eu me sinto humilhado	T3: Eu me sinto humilhado	Eu me sinto humilhado (a)	I feel humiliate	Eu me sinto humilhado (a)	Por causa da minha voz, eu me sinto humilhado (a).	

22	I feel depression	T1: Me sinto deprimido	T2: Sinto depressão	T3: Eu me sinto depressivo	Eu me sinto deprimido (a)	I feel depressed	Eu me sinto deprimido (a)	Por causa da minha voz, eu me sinto deprimido (a).	

23	I am more aggressive	T1: Estou mais agressivo (a)	T2: Eu estou mais agressivo	T3: Eu estou mais agressivo	Eu estou mais agressivo (a)	I am more aggressive	Eu estou mais agressivo (a)	Por causa da minha voz, eu estou mais agressivo (a).	

24	I am more fragile.	T1: Estou mais frágil	T2: Eu estou mais frágil	T3: Eu estou mais frágil	Eu estou mais frágil	I am more fragile	Eu estou mais frágil.	Por causa da minha voz, eu estou mais frágil.	

25	I feel inefficient.	T1: Me sinto ineficiente	T2: Eu me sinto ineficiente	T3: Eu me sinto ineficiente	Eu me sinto incapaz	I feel incapable	Eu me sinto incapaz	Por causa da minha voz, eu me sinto incapaz.	

26	I feel my opinions are not taken seriously by others.	T1: Sinto que os outros não levam minha opinião a sério	T2: Sinto que as minhas opiniões não são levadas a sério pelos outros	T3: Eu sinto que minha opinião não é levada a sério pelos outros	Eu sinto que os outros não levam minha opinião a sério	I feel the others do not consider my opinion seriously	Eu sinto que os outros não levam minha opinião a sério	Por causa da minha voz, eu sinto que os outros não levam as minhas opiniões a sério.	

27	Because of my voice, job performance was affected.	T1: Por causa da minha voz, minha performance no trabalho foi afetada	T2: Por causa da minha voz, minha performance no trabalho foi afetada	T3: Por causa da minha voz, o meu desempenho no trabalho foi afetado	O meu desempenho no trabalho foi afetado por causa da minha voz	My performance in work was affected	O meu desempenho no trabalho foi afetado por causa da minha voz.	Por causa da minha voz, meu desempenho no trabalho foi prejudicado.	

28	Because of my voice, I lost my job.	T1: Por causa da minha voz, perdi meu emprego	T2: Por causa da minha voz, eu perdi meu emprego	T3: Por causa da minha voz, eu perdi meu trabalho	Eu perdi meu emprego por causa da minha voz	I lost my job	Eu perdi meu emprego por causa da minha voz.	Por causa da minha voz, eu perdi meu trabalho.	

29	Because of my voice, I am earning less and it resulted in economic problems.	T1: Por causa da minha voz, estou ganhando menos dinheiro e isso me trouxe problemas econômicos	T2: Por causa da minha voz, minha renda diminuiu e isso me trouxe problemas econômicos	T3: Por causa da minha voz, estou ganhando menos e isso resultou em um problema econômico	Eu estou ganhando menos por causa da minha voz	I am earning less	Estou tendo prejuízo financeiro por causa da minha voz.	Por causa da minha voz, eu estou ganhando menos e tenho problemas financeiros.	

30	My job promotion encountered with problem.	T1: Minha promoção de trabalho não aconteceu	T2: Tive problemas para ser promovido	T3: Eu tive problemas para ser promovido no emprego	Eu tive problemas para ser promovido no emprego	I had trouble being promoted in my job	Eu tive problemas para ser promovido no emprego.	Por causa da minha voz, eu tive problemas para ser promovido no meu trabalho.	

31	Because of my voice, I lost many vocational positions.	T1: Por causa da minha voz, eu perdi muitas posições de trabalho	T2: Por causa da minha voz, perdi muitas posições de trabalho	T3: Por causa da minha voz, eu perdi muitas posições vocacionais	Eu perdi muitas oportunidades no meu trabalho por causa da minha voz	I lost job opportunities	Eu perdi oportunidades no meu trabalho por causa da minha voz.	Por causa da minha voz, eu perdi oportunidades no meu trabalho.	

32	Because of my voice, I dropped to low levels of job.	T1: Por causa da minha voz, meu cargo foi rebaixado	T2: Por causa da minha voz, minha posição de trabalho foi rebaixada	T3: Por causa da minha voz, eu diminuí meu nível de trabalho	Eu fui rebaixado no meu trabalho por causa da minha voz	I got demoted at my job	Eu fui rebaixado no meu trabalho por causa da minha voz.	Por causa da minha voz, eu fui rebaixado (a) no meu trabalho.	

33	I have trouble in communicating	T1: Tenho problemas para me comunicar	T2: Eu tenho dificuldade para me comunicar	T3: Eu tenho problemas para me comunicar	Eu tenho problemas para me comunicar	I have trouble communicating	Eu tenho problemas para me comunicar.	Por causa da minha voz, eu tenho problemas em me comunicar.	

34	I avoid speaking as much as possible.	T1: Faço de tudo para não falar	T2: Eu evito falar o máximo que posso	T3: Eu evito o máximo possível falar	Eu evito falar o máximo possível	I avoid speaking as most as I can	Eu evito falar o máximo possível.	Por causa da minha voz, eu evito falar sempre que possível.	

35	Others are misunderstood.	T1: Outros são mal compreendidos	T2: As pessoas não me compreendem bem	T3: Outros são mal compreendidos	As pessoas não me compreendem bem	People do not understand me well	As pessoas não me compreendem bem.	Por causa da minha voz, eu sou mal interpretado (a) pelos outros.	

36	Understandability of my voice is reduced.	T1: A compreensão da minha voz está reduzida	T2: A inteligibilidade da minha voz está reduzida	T3: A inteligibilidade a minha fala é reduzida	As pessoas têm dificuldade para entender o que eu falo	People have trouble in understanding what I say	As pessoas têm dificuldade para entender o que eu falo.	Por causa da minha voz, os outros têm dificuldades em entender o que eu falo.	

37	I am indifferent to asking in various statuses.	T1: Não faço mais perguntas	T2: Estou indiferente para questionar em várias situações	T3: Eu estou indiferente para perguntas em vários status	Eu evito fazer perguntas em várias situações	I avoid making questions in many situations	Eu evito fazer perguntas em várias situações.	Por causa da minha voz, eu evito fazer perguntas em várias situações.	

38	People ask me to speak louder.	T1: As pessoas me pedem para falar mais alto	T2: As pessoas pedem para eu falar mais alto	T3: As pessoas me pedem para falar com inteligibilidade	As pessoas pedem para eu falar mais claro	People ask me to speak louder	As pessoas pedem para eu falar mais alto.	Por causa da minha voz, as pessoas pedem para eu falar mais alto.	

39	People request me to speak more intelligibly.	T1: As pessoas me pedem para falar melhor	T2: As pessoas pedem para eu falar mais claro	T3: As pessoas me pedem para falar com inteligibilidade	As pessoas pedem para eu falar mais claro	People ask me to speak clearer	As pessoas pedem para eu falar mais claro.	Por causa da minha voz, as pessoas pedem para eu falar mais claro.	

40	I have trouble communicating in noisy environments.	T1: Tenho dificuldade em me comunicar em locais barulhentos	T2: Eu tenho problemas para me comunicar em ambientes ruidosos	T3: Eu tenho dificuldades para me comunicar em locais barulhentos	Eu tenho dificuldade para falar em locais barulhentos	I have trouble when speaking in noisy environments	Eu tenho dificuldade para falar em locais barulhentos.	Por causa da minha voz, eu tenho dificuldades de falar em locais barulhentos.	

41	I have trouble in participation in social activities.	T1: Tenho problemas para participar de atividades sociais	T2: Eu tenho problemas para participar de atividades sociais	T3: Eu tenho problemas para participar de atividades sociais	Eu tenho problemas para participar de atividades sociais	I have problems participating in social activities	Eu tenho problemas para participar de atividades sociais.	Por causa da minha voz, eu tenho problemas para participar de atividades sociais.	

42	I avoid of speaking in public.	T1: Evito falar em público	T2: Eu evito falar em público	T3: Eu evito falar em público	Eu evito falar em público	I avoid speaking in public	Eu evito falar em público.	Por causa da minha voz, eu evito falar em público.	

43	My partnership in family decisions is decreased.	T1: Minha participação nas decisões familiares diminuiu	T2: A minha participação nas decisões da família diminuiu	T3: Minha participação nas decisões familiares diminuiu	Eu participo menos nas decisões da minha família	I participate less in my family’s decisions	Eu participo menos nas decisões da minha família.	Por causa da minha voz, eu participo menos das decisões familiares.	


**Legenda:** T1 = tradutor inglês-português 1; T2 = tradutor inglês-português 2; T3 = tradutor inglês-português 3; Síntese = versão em português da compilação das traduções do T1 + T2 + T3

Após a retrotradução, na análise do comitê de especialistas, foram necessários ajustes em 15 itens: 4, 5, 10, 13, 14, 17, 27, 28, 29, 30, 33, 34, 35, 38 e 43. O pronome “eu” foi acrescentado em todas as afirmações para evitar dúvida ou erro de interpretação. No instrumento original, “Por causa da minha voz” vinha descrita somente antes dos itens 6 e 33. Para facilitar a compreensão e evitar erro ao interpretar as afirmativas, o comitê de especialistas incluiu “Por causa da minha voz” no início dos itens 6 a 43.

Participaram do pré-teste 36 adultos na faixa de risco para distúrbios vocais, com idade média de 39,39±13,15 anos, sendo 25 do sexo feminino e 11 do sexo masculino. Dos participantes, 32 (88,89%) possuíam queixa vocal e 4 (11,11%) não possuíam.

No pré-teste, a opção “não aplicável” foi marcada nos itens 4, 5, 6, 7, 12, 13, 14, 18, 32 e 34. Porém, a proporção de marcação da opção não aplicável foi significativamente menor que a das opções da chave de resposta tradicional para todos os itens do instrumento no pré-teste (Tabela 1).

**Tabela 1:** Análise do pré-teste na população-alvo

Item	NA		Chave de resposta tradicional		p-valor
	n	%	n	%	
Eu tenho problemas para falar muito ao telefone	0	0	36	100	1
Eu tenho problemas para falar alto	0	0	36	100	1
Eu tenho dor de cabeça quando eu falo	0	0	36	100	1
Eu tenho problemas para manter a minha voz quando eu falo	2	5,56	34	94,44	0,002
A minha voz às vezes está boa e às vezes ruim	0	0	36	100	1
Por causa da minha voz, eu fico nervoso (a)	1	2,78	35	97,22	<0,001
Por causa da minha voz, eu sinto que incomodo os outros	0	0	36	100	1
Por causa da minha voz, eu tenho dificuldades para fazer amigos	0	0	36	100	1
Por causa da minha voz, eu perdi a autoconfiança	0	0	36	100	1
Por causa da minha voz, eu tenho problemas para expressar quem eu sou	0	0	36	100	1
Por causa da minha voz, eu tenho problemas para expressar minhas ideias	0	0	36	100	1
Por causa da minha voz, eu fico com vergonha	0	0	36	100	1
Por causa da minha voz, eu perco a calma	0	0	36	100	1
Por causa da minha voz, eu sou ridicularizado (a)	0	0	36	100	1
Por causa da minha voz, eu me sinto rejeitado (a) e sozinho (a)	0	0	36	100	1
Por causa da minha voz, eu me sinto frustrado (a)	0	0	36	100	1
Por causa da minha voz, eu evito responder aos outros	0	0	36	100	1
Por causa da minha voz, eu me sinto ansioso (a)	1	2,78	35	97,22	<0,001
Por causa da minha voz, eu não tenho esperança no futuro	0	0	36	100	1
Por causa da minha voz, eu congelo quando falo	0	0	36	100	1
Por causa da minha voz, eu me sinto humilhado (a)	0	0	36	100	1
Por causa da minha voz, eu me sinto deprimido (a)	0	0	36	100	1
Por causa da minha voz, eu estou mais agressivo (a)	0	0	36	100	1
Por causa da minha voz, eu estou mais frágil	0	0	36	100	1
Por causa da minha voz, eu me sinto incapaz	0	0	36	100	1
Por causa da minha voz, eu sinto que os outros não levam as minhas opiniões a sério	0	0	36	100	1
Por causa da minha voz, meu desempenho no trabalho foi prejudicado	0	0	36	100	1
Por causa da minha voz, eu perdi meu trabalho	0	0	36	100	1
Por causa da minha voz, eu estou ganhando menos e tenho problemas financeiros	0	0	36	100	1
Por causa da minha voz, eu tive problemas para ser promovido no meu trabalho	0	0	36	100	1
Por causa da minha voz, eu perdi oportunidades no meu trabalho	0	0	36	100	1
Por causa da minha voz, eu fui rebaixado (a) no meu trabalho	1	2,78	35	97,22	<0,001
Por causa da minha voz, eu tenho problemas em me comunicar	0	0	36	100	1
Por causa da minha voz, eu evito falar sempre que possível	1	2,78	35	97,22	<0,001
Por causa da minha voz, eu sou mal interpretado (a) pelos outros	0	0	36	100	1
Por causa da minha voz, os outros têm dificuldades em entender o que eu falo	0	0	36	100	1
Por causa da minha voz, eu evito fazer perguntas em várias situações	0	0	36	100	1
Por causa da minha voz, as pessoas pedem para eu falar mais alto	0	0	36	100	1
Por causa da minha voz, as pessoas pedem para eu falar mais claro	0	0	36	100	1
Por causa da minha voz, eu tenho dificuldades de falar em locais barulhentos	0	0	36	100	1
Por causa da minha voz, eu tenho problemas para participar de atividades sociais	0	0	36	100	1
Por causa da minha voz, eu evito falar em público	0	0	36	100	1
Por causa da minha voz, eu participo menos das decisões familiares	0	0	36	100	1

Teste de igualdade de duas proporções

Legenda: NA = não aplicável

A versão final do IVQLP traduzida e adaptada para o PB foi denominada de IVQLP-Br (Anexo A), apresenta 43 itens, e uma chave de respostas de quatro níveis.

## DISCUSSÃO

Os protocolos de autoavaliação têm sido muito utilizados na prática clínica na área da saúde a fim de compreender o impacto de um possível problema vocal na perspectiva do paciente, além de ajudar a estimar sua adesão ao tratamento^(2,5,7,11,14-16)^.

A adaptação transcultural dos instrumentos de autoavaliação tem em vista a adequação para sua aplicabilidade na população desejada. Por meio dela é possível que as diferenças socioculturais entre as culturas e os idiomas sejam resolvidas, não sendo, dessa forma, uma mera tradução. A adaptação e posterior validação do instrumento tem sido muito utilizada nas áreas da Fonoaudiologia para fornecer instrumentos com boas propriedades psicométricas em português brasileiro para a clínica vocal^(15,16)^.

O IVQLP, protocolo originalmente iraniano, já utilizado na língua inglesa, tem se destacado por ser um instrumento mais aprofundado que os instrumentos já validados para o português brasileiro. Provavelmente isso ocorre porque ele traz uma análise de um número maior de domínios relacionados a qualidade de vida em voz. Ele é composto por itens dos domínios físico,emocional e funcional, sendo que esse último se subdivide ainda em três subescalas, funcional em relação ao trabalho, comunicação diária e atividades sociais do paciente. É possível observar que o IVQLP aprofunda-se na relação voz x emocional e voz x relação com o trabalho, como vemos na questão 25 por exemplo, que diz “por causa da minha voz, eu me sinto incapaz”, ou ainda na questão 30, “por causa da minha voz, eu tive problemas para ser promovido no meu trabalho”, buscando reconhecer de forma aprofundada o impacto da voz nos diferentes domínios da vida dos indivíduos, o que contribui para um planejamento terapêutico direcionado às necessidades do paciente. Além disso, parece ser um interessante instrumento a ser usado nas pesquisas com os falantes do Português Brasileiro.

Para a tradução e adaptação transcultural do instrumento para o português brasileiro, três fonoaudiólogos especialistas em voz e fluentes no inglês, realizaram traduções do mesmo. A partir disso, uma versão de síntese foi elaborada e os próprios autores resolveram as discordâncias existentes entre si. Em seguida, foi realizada a retrotradução, em que o comitê de especialistas, necessitou ajustar 15 itens, além de acrescentar a frase “Por causa da minha voz” antes das questões de 6 a 43, a fim de deixar as afirmações mais claras, evitando assim, dúvidas ou erros de interpretação.

No pré-teste foi observado que a opção de resposta “não aplicável” foi assinalada doze vezes, sendo 2 vezes nos itens 4 e 6, e uma vez nos itens 5, 7, 12, 13, 14, 18, 32 e 34. Porém, esses números não obtiveram proporção significativamente maior que as opções da chave de resposta tradicional para nenhum dos itens do instrumento no pré-teste, portanto nenhuma questão precisou ser modificada. Entende-se que, de um modo geral, a população-alvo compreendeu os 43 itens, e que eles são aplicáveis no PB.

Dessa forma, foi possível produzir uma versão adaptada transculturalmente para o PB do IVQLP^(10,11)^, chamada IVQLP-Br. Entendemos que esse instrumento possa contribuir na clínica vocal, acrescentando informações úteis para o manejo do caso. Embora mais longo que outros questionários disponíveis para a clínica vocal, como o IDV^(14)^ e o QVV^(5)^, é mais abrangente que este último, o único que aborda o conceito de qualidade de vida, até então. Contudo, o presente estudo ateve-se apenas à adaptação transcultural e é necessário a continuidade do estudo com a realização da validação para demonstrar as propriedades psicométricas do instrumento e comprovar que o IVQLP-Br é válido, confiável e tem boa acurácia diagnóstica em PB.

## CONCLUSÃO

Conclui-se que a versão traduzida do IVQLP para o PB, denominada IVQLP-Br, apresentou equivalência transcultural, e, após todas as etapas da validação serem concluídas, poderá ser utilizada para uma observação mais aprofundada dos impactos da voz nos diferentes domínios da vida dos indivíduos, podendo contribuir tanto para a prática clínica, quanto para pesquisas com falantes do PB.

## Apêndice A QUESTIONÁRIO

Sexo: ( ) Masculino ( ) Feminino

Idade:

Possui queixa vocal: ( ) Sim ( ) Não

Possui alterações neurológicas: ( ) Sim ( ) Não

Possui alterações cognitivas: ( ) Sim ( ) Não

## Anexo A. Perfil de qualidade de vida relacionado à voz (IVQLP-Br)

Responda às seguintes perguntas dependendo do tamanho do seu problema. Use a seguinte escala:

1 = nenhum, não é um problema

2 = é um problema pequeno

3 = é um problema moderado

4 = é um problema grande

**Table:**

1.	Eu tenho problemas para falar muito ao telefone	1	2	3	4
2.	Eu tenho problemas para falar alto	1	2	3	4
3.	Eu tenho dor de cabeça quando eu falo	1	2	3	4
4.	Eu tenho problemas para manter a minha voz quando eu falo	1	2	3	4
5.	A minha voz às vezes está boa e às vezes ruim	1	2	3	4
6.	Por causa da minha voz, eu fico nervoso (a)	1	2	3	4
7.	Por causa da minha voz, eu sinto que incomodo os outros	1	2	3	4
8.	Por causa da minha voz, eu tenho dificuldades para fazer amigos	1	2	3	4
9.	Por causa da minha voz, eu perdi a autoconfiança	1	2	3	4
10.	Por causa da minha voz, eu tenho problemas para expressar quem eu sou	1	2	3	4
11.	Por causa da minha voz, eu tenho problemas para expressar minhas ideias	1	2	3	4
12.	Por causa da minha voz, eu fico com vergonha	1	2	3	4
13.	Por causa da minha voz, eu perco a calma	1	2	3	4
14.	Por causa da minha voz, eu sou ridicularizado (a)	1	2	3	4
15.	Por causa da minha voz, eu me sinto rejeitado (a) e sozinho (a)	1	2	3	4
16.	Por causa da minha voz, eu me sinto frustrado (a)	1	2	3	4
17.	Por causa da minha voz, eu evito responder aos outros	1	2	3	4
18.	Por causa da minha voz, eu me sinto ansioso (a)	1	2	3	4
19.	Por causa da minha voz, eu não tenho esperança no futuro	1	2	3	4
20.	Por causa da minha voz, eu congelo quando falo	1	2	3	4
21.	Por causa da minha voz, eu me sinto humilhado (a)	1	2	3	4
22.	Por causa da minha voz, eu me sinto deprimido (a)	1	2	3	4
23.	Por causa da minha voz, eu estou mais agressivo (a)	1	2	3	4
24.	Por causa da minha voz, eu estou mais frágil	1	2	3	4
25.	Por causa da minha voz, eu me sinto incapaz	1	2	3	4
26.	Por causa da minha voz, eu sinto que os outros não levam as minhas opiniões a sério	1	2	3	4
27.	Por causa da minha voz, meu desempenho no trabalho foi prejudicado	1	2	3	4
28.	Por causa da minha voz, eu perdi meu trabalho	1	2	3	4
29.	Por causa da minha voz, eu estou ganhando menos e tenho problemas financeiros	1	2	3	4
30.	Por causa da minha voz, eu tive problemas para ser promovido no meu trabalho	1	2	3	4
31.	Por causa da minha voz, eu perdi oportunidades no meu trabalho	1	2	3	4
32.	Por causa da minha voz, eu fui rebaixado (a) no meu trabalho	1	2	3	4
33.	Por causa da minha voz, eu tenho problemas em me comunicar	1	2	3	4
34.	Por causa da minha voz, eu evito falar sempre que possível	1	2	3	4
35.	Por causa da minha voz, eu sou mal interpretado (a) pelos outros	1	2	3	4
36.	Por causa da minha voz, os outros têm dificuldades em entender o que eu falo	1	2	3	4
37.	Por causa da minha voz, eu evito fazer perguntas em várias situações	1	2	3	4
38.	Por causa da minha voz, as pessoas pedem para eu falar mais alto	1	2	3	4
39.	Por causa da minha voz, as pessoas pedem para eu falar mais claro	1	2	3	4
40.	Por causa da minha voz, eu tenho dificuldades de falar em locais barulhentos	1	2	3	4
41.	Por causa da minha voz, eu tenho problemas para participar de atividades sociais	1	2	3	4
42.	Por causa da minha voz, eu evito falar em público	1	2	3	4
43.	Por causa da minha voz, eu participo menos das decisões familiares	1	2	3	4
